# Did the introduction of pictorial health warnings increase information seeking for smoking cessation?: Time-series analysis of Google Trends data in six countries

**DOI:** 10.18332/tpc/111130

**Published:** 2019-08-02

**Authors:** Anton E. Kunst, Casper van Splunter, Sigrid A. Troelstra, Jizzo R. Bosdriesz

**Affiliations:** 1 Department of Public Health, Amsterdam UMC, University of Amsterdam, Amsterdam Public Health Research Institute, Amsterdam, The Netherlands

**Keywords:** Google Trends research, smoking cessation, online information seeking, public health warnings, tobacco control, Europe

## Abstract

**INTRODUCTION:**

Pictorial health warnings (PHW) can influence smoking cessation rates and precursors thereof. However, both the magnitude and duration of their impact, in national populations, remain uncertain because of limitations of the available data. In this study we used Google Trends data from six European countries to evaluate whether the implementation of PHW was followed by a short-term increase in online searches on smoking cessation.

**METHODS:**

We applied an interrupted time-series design using ARIMA models. We used weekly or monthly data on the relative search volume (RSV) for search terms about smoking cessation. First, RSV trends were seasonally adjusted and adjusted for autocorrelation. Next, regression models were fitted that included terms for the potential effect of PHW in month 1, months 2–3, and months 4–6 after implementation.

**RESULTS:**

Our findings for France and the United Kingdom were partly in line with our initial expectations. In France, a 4% increase (95% CI: -2% – 11%) occurred in the first month after implementation, but not later. In the UK, a 3% increase (95% CI: 1% – 6%) in ‘quit smoking’ searches occurred in months 2–3. No increases were observed for any other periods for France, the UK, Ireland, Norway, Denmark or Switzerland.

**CONCLUSIONS:**

We found no consistent support that the implementation of PHW was associated with increased internet searches for smoking cessation. Further studies are needed to assess and understand the magnitude and duration of population-wide impacts of PHW.

## INTRODUCTION

The urgent need for further action in tobacco control derives from irrefutable evidence on the harmful effects of smoking^1-4^ combined with perplexing numbers of deaths attributable to tobacco use. Tobacco use is estimated to lead to around 6 million deaths worldwide each year, a number that could rise to 8 million deaths by 2030. At least half of all current tobacco users are likely to die from a tobacco related disease^1^.

The fight against tobacco use in Europe has been ongoing for decades, and is still expanding. The MPOWER project of the World Health Organization identified a large number of potentially effective policy measures, including taxation of tobacco, banning advertisements, restricting smoking in public places, and supporting people who want to quit^5^. One measure that is introduced in increasingly more countries, including the member states of the European Union, is pictorial health warnings (PHW) on cigarette packages^6^. As of 2014, PHW were implemented in 77 countries or jurisdictions that together covered half of the world population^7^.

PHW are in place as a means to disseminate information on the health hazards of smoking; this continues to be relevant given the fact that much of the world’s population is still not fully aware of most of these hazards^8^. Common gaps in this awareness include limited knowledge of health consequences apart from the most obvious ones such as lung cancer risk^9^, poor understanding of the addictive and toxic nature of tobacco^10^, in addition to a general tendency to negate or disengage from information regarding health hazards^11^. Yet, awareness and acceptance of such information is found to increase a smoker’s chance to give up smoking^12^.

Several studies have aimed to assess the effectiveness of PHW in increasing cessation rates^13-20^. Most of these studies had an experimental design. A review from 2011 concluded that PHW had a positive impact on smokers’ tendency to consider quitting smoking, and that this effect depended on the size and design of the pictures^13^. One study found that PHW that elicit strong emotional reactions are significantly more effective in making people consider quitting^14^. Another study concluded that PHW are more effective if they are placed within a social context, referring to the hazardous effects of passive smoking^20^. Unfortunately, according to a 2014 review, the quality of the published studies was mostly poor to very poor, thus affecting the strength of the available evidence^16,21^. This implied a need for stronger methodological designs with prospective follow-up^16^, such as a more recent trial that supported earlier evidence on the effectiveness of PHW^17^.

A common limitation of these experimental studies is that results of the introduction of PHW among national populations cannot be readily generalized to the real-world situation^21^. Complementary evidence can come from studies that assess the effects of the introduction of PHW in real-world settings. A study of 27 European countries suggested that PHW were positively associated with smoking cessation behaviour^18^. Both textual and pictorial warnings on cigarette packages were associated with cognitive and behavioural outcomes, with stronger associations for pictorial warnings. An evaluation of changes in health warnings on cigarette packages between 2002 and 2011 in Canada and the USA concluded that the PHW implemented in Canada had greater effect than the text-only warnings in the United States^19^. A review of longitudinal observational studies from 20 countries found that knowledge of the health effects of smoking increased significantly after introduction of PHW, as did the number of quit attempts^22^. In addition, studies reported a significant but modest decrease in the smoking prevalence after introduction of PHW.

In this paper, we aim to generate further evidence on the potential impact of the real-world introduction of PHW. We apply a time-series design in which trends in smoking cessation behaviours are assessed around the time of the introduction of PHW in six European countries. For this, we use data from Google Trends, a data source that has been used to perform time-series analyses in various outcomes^23,24^ including the use of tobacco products^25,26^. USA studies showed that Google Trends data are a valuable tool for monitoring tobacco use^26^, including cigar use and smokeless tobacco^25^. A Dutch study using Google Trends found that online information seeking regarding smoking cessation increased after the introduction of a national ban on smoking in bars and reimbursement for smoking cessation services^27^.

The general aim of this study was to investigate whether the national implementation of PHW was followed by an increase in online information seeking behaviour on smoking cessation. We assumed that the online search rate would reflect the number of people within a country that is considering to quit smoking. Based on this assumption, we expected that the introduction of PHW, if effective in changing smoking cessation behaviour in the short-term, would be followed by an increase in the online search rate for smoking cessation. We tested this expectation for six different European countries, as the occurrence of a detectable change could depend on national conditions and the precise way in which countries introduce the PHW.

## METHODS

### Data

For this study we used Google Trends data, extracted in March 2015. Google expresses the search rate as the relative search volume (RSV), which is used for assessing trends in the relative search volume over time, in place of the absolute number of search queries made. To calculate the RSV, Google looks at the number of times that the requested search term has been searched during each week over the period and country selected by the user. The highest peak in this number of searches is given the value of 100, and the other values are scaled relative to this reference value. Thus, a value of 50 would correspond to a search volume half as high as the highest peak value. We obtained these data for each country separately, which means that the highest peak within each country is set to 100. We only use these data to assess changes over time within countries, not to study differences between countries (which the Google Trends interface does allow).

We selected European countries where PHW were implemented before 2014, in order to have sufficient time for follow-up analysis. From these countries, we excluded Latvia, Malta, Romania, Spain and Ukraine because less than 80% of the total population had access to internet^28^. Moreover, Belgium had to be excluded due to a gap in the Google Trends data immediately after the implementation of the PHW. The six remaining countries are given in Table 1, together with the dates of implementation of the PHW. In all countries, about 50 per cent of the packaging area of cigarette packages was covered by PHW at the time of their introduction. For Ireland, Norway and Switzerland, search volumes per week were too low to be provided, therefore for these countries, we could only access the RSV volume per month.

**Table 1:** The Implementation of PHW in different European countries

*Country*	*Implementation deadline (formal start)*	*Coverage % (front-back)*	*Search term used*
Denmark	February 2012 (February 2011)	35 (30 – 40)	Rygestop
France	April 2012 (April 2010)	53 (- – 53)	Arreter de fumer
Ireland	February 2013 (December 2011)	52 (45 – 58)	Stop smoking
Norway	July 2011 (October 2009)	48 (43 – 53)	Røykeslutt
Switzerland	January 2010 (January 2010)	56 (48 – 63)	Rauchstopp
United Kingdom	September 2010 (October 2008)	53 (- – 53)	Stop smoking, Quit smoking

PHW: pictorial health warnings.

For each country, we selected a search term in the main local language that was equivalent to ‘stop smoking’ (Table 1). If various terms could be used, we selected the term used most frequently in that country. For the United Kingdom, we selected two search terms, ‘stop smoking’ and ‘quit smoking’, because both terms were frequently used. For Ireland, ‘stop smoking’ was used more frequently. Therefore, only this search term was selected.

Google Trends data count searches not users. As a result, no distinction can be made according to user sociodemographic characteristics such as age and sex. Google Trends data include every search made within that country, except for repeated searches that a user makes within a short period of time.

### Statistical analyses

The unadjusted relative search volumes for smoking cessation show regularly occurring peaks in search queries, such as in the first week of January, when many people tend to make New Year’s resolutions and attempt to quit smoking. Therefore, to avoid attributing an intervention effect to a seasonally occurring peak or other season-bound changes, a seasonal decomposition was performed. The seasonally adjusted series were log-ransformed in order to approximate a normal distribution of the variables and to stabilize variances over time.

We analysed the adjusted data using autoregressive integrated moving average (ARIMA) modelling, for each search term and country separately. Using ARIMA enabled us to account for dependence between the data points in the time-series, which would otherwise cause standard errors to be estimated incorrectly. The Box-Jenkins method, which consists of model identification, parameter estimation and model checking, was applied in order to find the best fitting time-series model^29^. Even though the regression method could include both auto-regression, integration, and a moving average, some of these components can be left out in the final model. The final model specification was determined for each country based on which model best fitted the data. The parameters used in the final models for each country are given in Table 2. Similar ARIMA model specifications were used in each country, with the exception of the UK (‘stop smoking’) and Norway.

**Table 2:** The best fitting ARIMA model parameters per country and interval of data used per country

*Country*	*Model parameters^a^*	*Data interval*
Denmark	0,1,1 (1,0,0)	Weekly
France	0,1,1 (0,0,0)	Weekly
Ireland	0,1,1 (0,0,0)	Monthly
Norway	1,0,0 (0,0,0)	Monthly
Switzerland	0,1,1 (0,0,0)	Monthly
UK (stop smoking)	1,1,9 (0,0,0)	Weekly
UK (quit smoking)	0,1,1 (1,0,1)	Weekly

a These are the p, d and q parameters of the best fitting ARIMA model, with the seasonal parameters inside brackets.

This final model was used to estimate the association with the implementation of PHW (Table 1). To assess the timing and duration of the potential effect of PHW, three effect periods were distinguished: 1 month, 2–3 months and 4–6 months after implementation. A more detailed distinction was not included, in order to avoid the risks of multiple testing. The respective periods were modelled as binary intervention dummies coded ‘1’ for the duration of the intervention period and coded ‘0’ for other weeks. The dummies for the different periods were added simultaneously to the final model. In addition, the model controlled for long-term trends, measured by numbering all weeks from 1 onwards. Output was measured in percentage points relative to the expected level without the influence of the introduction of PHW. All analyses were performed in IBM SPSS (version 21).

Given that the applied ARIMA models include several components in order to account for dependence between the data points, they may run the risk of removing variations that may have resulted from PHW implementation. To check for such potential over-adjustment, we performed a series of sensitivity analyses. First, we re-ran all analyses with the ARIMA model (0,1,1) (0,0,0), which is a commonly used parametrization and makes the models easier to compare (Table 4). Secondly, we re-ran the analysis for all countries using the data without log-transformation and the same (0,1,1) (0,0,0) ARIMA parameters (Appendix Table 1). Both sensitivity analyses yielded the same key results reported below. Finally, we applied an ARIMA model without any adjustment for autoregression, integration, or moving average and no log-transformation (0,0,0) (0,0,0), only with a seasonal decomposition (Appendix Table 2). This latter analysis showed, not unexpectedly, greater discrepancies in results, and particularly wider 95% confidence intervals. However, similarly to what is reported below, it did not yield consistent evidence regarding trends around the introduction of PHW.

**Table 3:** Effect of the implementation of PHW on the RSV in three periods with the best fitting ARIMA model per country

*Country*	*1 month after implementation*		*2–3 months after implementation*		*4–6 months after implementation*	
	*RoC^a^*	*(95% CI)*	*RoC*	*(95% CI)*	*RoC*	*(95% CI)*
Denmark	1.02	0.97–1.06	1.01	0.98–1.04	1.01	0.98–1.03
France	1.04	0.98–1.11	1.02	0.97–1.06	0.99	0.95–1.03
Ireland	1.01	0.92–1.11	0.99	0.93–1.05	1.01	0.96–1.06
Norway	0.90	0.79–1.02	0.96	0.87–1.07	1.02	0.93–1.11
Switzerland	0.96	0.83–1.11	0.94	0.85–1.04	1.01	0.92–1.10
UK Stop^b^	1.00	0.92–1.08	1.01	0.95–1.07	1.00	0.95–1.04
UK Quit^b^	1.01	0.97–1.05	**1.03**	**1.01–1.06**	0.99	0.97–1.01

RSV: relative search volume. PHW: pictorial health warnings. RoC: ratio of change.

a Estimated RoC in RSV due to the introduction of PHW.

b For the UK, two different search terms were modelled separately: ‘Stop smoking’, and ‘Quit smoking’. Significant effects are highlighted in bold (p<0.05).

**Table 4:** Sensitivity analysis: effect of the implementation of PHW on the RSV in three periods with a standard ARIMA model (0,1,1) (0,0,0) per country

*Country*	*1 month after implementation*		*2–3 months after implementation*		*4–6 months after Implementation*	
	*RoC^b^*	*(95% CI)*	*RoC*	*(95% CI)*	*RoC*	*(95% CI)*
Denmark	1.02	0.97–1.06	1.01	0.98–1.04	1.01	0.98–1.03
Norway	0.92	0.80–1.04	1.03	0.95–1.12	1.03	0.99–1.07
UK Stopc	1.00	0.95–1.05	1.01	0.98–1.04	1.00	0.97–1.02
UK Quitc	1.01	0.96–1.07	1.03	0.99–1.07	0.99	0.96–1.02

a France, Ireland and Switzerland were not included in the sensitivity analysis because the best fitting ARIMA model for these countries is the same as the standard ARIMA model.

b Estimated ratio of change (RoC) in RSV due to the introduction of PHW.

c For the UK, two different search terms were modelled separately: ‘Stop smoking’, and ‘Quit smoking’. Significant effects are highlighted in bold (p<0.05).

## RESULTS

The detailed RSV trends per country are incorporated in the Supplementary files (S1–S7). These figures display both the trends observed after seasonal adjustment and the trends after adjustment using the ARIMA models as specified in Table 2. In many countries, the RSV strongly varies over time. In France, the RSV increased shortly after implementation of the PHW. In other countries, no such immediate increase is visible from the graphs, in some countries the RSV shows a slight decrease after the introduction of PHW.

Table 3 shows the results of the regression analyses of the potential effect of PHW. The ratios represent the ratio of change in RSV in a period after the implementation of PHW compared to the baseline value. In the United Kingdom, estimates for the two search terms followed the same pattern, with a small increase in months 1 and 2–3 followed by a small decrease 4–6 months after implementation. The increase in months 2–3 for ‘quit smoking’ was statistically significant and implied an increase of 3% in RSV after the implementation of PHW. A similar pattern was observed in France, where the RSV increased by 4% in month 1, but decreased by 1% in months 4–6. However, none of these changes was statistically significant. In other countries, we found no consistent increases to support our hypothesis. In Denmark and Ireland, changes were small. In Norway and Switzerland, the RSV decreased in the first month after implementation, and in Norway, this decrease was large in absolute terms. However, none of these changes was statistically significant.

Table 4 presents the results of the sensitivity analysis using ARIMA models with a standard (0,1,1) parametrization. France, Ireland and Switzerland were not included because the best fitting ARIMA model for these countries was the same as the standard ARIMA model used for the sensitivity analysis. Results for the other countries are very similar to those presented in Table 3, only the significance levels varied slightly. The main differences were that the effect for the UK (‘quit smoking’) was no longer significant, while the effect for Norway at 2–3 months came close to being significant.

Appendix Table A1 shows the results for the sensitivity analysis using non log-transformed data and a standard ARIMA parametrization. Because these data are non-transformed, they read as a beta coefficient rather than a ratio. The effect for France, 1 month after implementation, is still observed but now with statistical significance. Conversely, the significant effect for the UK disappears. In general, the pattern of results is similar, with most effects estimates being small and not statistically significant.

## DISCUSSION

Various studies have shown that PHW may influence the perception, beliefs and knowledge about tobacco products and the risks associated with use of these products. Through these precursors of smoking, PHW may influence smoking initiation and cessation rates^21^. Population studies have suggested modest impacts on population-wide trends in smoking cessation and smoking prevalence. However, due to methodological limitations, the magnitude and timing of these real-world impacts remain uncertain.

The aim of this study was to contribute to the evidence base by evaluating the implementation of PHW in six European countries, using an interrupted time-series design with weekly or monthly data on internet searches for smoking cessation. We expected the implementation of PHW to be followed by a short-term increase in searches for online information on smoking cessation in six European countries. Results for France and the United Kingdom were partly in line with our initial expectations. However, the increases in France were not statistically significant, whereas significant increases occurred in the UK only in ‘quit smoking’ searches (not in ‘stop smoking’) and only 2–3 months after implementation of PHW. Moreover, no (significant) increases were observed in the four other countries.

### Evaluation of methodology

Interrupted time-series design can be used to gain moderate to strong evidence for the impact of a policy measure that is introduced at a single point in time. The potential of Google Trends data for such an assessment was shown in a previous analysis on The Netherlands^27^. This showed clear short-term changes in internet searches for smoking cessation after the introduction of a ban on smoking in bars and restaurants and reimbursement of smoking cessation services.

A potential limitation of the current application is that there was no single date of implementation of the PHW. In every country, except Switzerland, the deadline of the implementation of PHW was preceded by a period during which the PHW could be introduced (Table 1). We assumed that the population exposure to PHW increased slowly before the deadline, but became especially rapid at the time of the implementation deadline. We checked this assumption by assessing trends in Google Trends data on search terms similar to ‘pictures on cigarette packages’. Supplementary Figure S8 shows trends for Denmark, France, and the UK (no relevant data could be extracted from Google Trends for Ireland, Norway and Switzerland). In each country, the highest peaks in RSV occur exactly on the dates of the implementation deadlines, supporting our assumption of maximal population-wide exposure around these dates.

Whereas we had weekly data for UK, France, and Denmark, data for the other countries were only available per month. Monthly data did not affect our measurement to PHW exposure because we distinguished monthly exposure periods, and because implementation dates coincided with the first day of a month. However, control for underlying RSV trends could not be as accurate with monthly data as with weekly data. Perhaps this contributed to the fact that no evidence for a positive impact of PHW implementation was found for any of the countries with monthly data.

We used ARIMA modelling to control for potential confounding by trends that occur gradually. Inspection of Supplementary Figures S1–S7, however, shows important irregularities in RSV trends. These irregularities may reflect the impact of specific events such as public information campaigns, sudden price increases or other national events. We could not identify and control for all potentially relevant events in all six countries. Possibly, such explicit control for events would have led to a better model fit, and therefore a more accurate assessment of the impact of the implementation of PHW.

Using Google Trends data has some notable advantages, such as being able to study a precursor of behavioural change that is not covered by traditional smoking behaviour surveys. Searching for information online offers very low barriers, it is accessible to most people, comes with no additional costs, and is virtually anonymous. For many people, the internet is now the first source of information regarding their health, and the information found online might even be more readily accepted than the advice of health professionals^30^. In addition, having weekly data provides a level of detail not commonly found in interview surveys. This time detail allowed us to accurately assess the timing and duration of hypothesized effects. This analytical potential is illustrated in previous applications of this method, where we were able to demonstrate short-term and medium-term effects of, respectively, smoke-free legislation, reimbursement of stop-smoking medication, and a national smoking cessation campaign^27,31^.

On the other hand, using Google Trends data has some specific limitations. Not everyone uses Google when they search for online information. However, for the countries and periods in this study, Google’s market share ranged between 90% and 98%^32^. Additionally, an important consideration when using internet search volume as an outcome is that many of these searches may not have led to real-life action, such as a quit attempt. Therefore, these data are valid for looking at changes in precursors of behaviour at the population level, but not studying behaviour change at the individual level.

PHW can have a different impact in different subgroups of the population, in terms of age, gender and educational group^33-35^. Unfortunately, with Google Trends data, we could not distinguish between such subgroups and we might therefore have missed specific effects for some subgroups. Since the data are completely anonymous and untraceable to individual users, it is hard to ascertain sociodemographic characteristics in the study sample. It is known that younger people, and those with a higher education and/or higher income use the internet more frequently^36^. However, older adults might be as likely as adolescents to use online search engines such as Google to look for health related information^37^.

Another factor to consider is the duration of potential effects, as other studies of effects on internet search volumes have shown quite short-lived effects^27,31^. This means that even with weekly data, it might be hard to identify potential effects, and even more so with monthly data.

### Interpretation of results

In some countries we observed substantial short-term fluctuations in internet searching for smoking, even after correcting for seasonal trends. The causes of these fluctuations are unknown. Possibly, they reflect group behaviour driven by collective processes that involve social interactions, emotion, and curiosity. After applying our statistical models, we found no clear evidence that PHW increase internet searching for smoking cessation related information. This lack of demonstrable impact might be related to avoidance behaviour among regular smokers. A study on regular smokers involved in the International Tobacco Control (ITC) project found that the implementation of PHW in the UK and France increased warning avoidance, but did not increase warning salience^38^. A UK study found that the PHW increased warning avoidance by regular smokers. In contrast, they showed no increase in warning salience or warning persuasiveness^39^. The lack of such increases in warning salience could explain a lack in increase of internet searching for smoking cessation.

Our findings are in contrast to population-wide studies that suggest that PHW are associated with modest increases in self-reported intention to quit, or actual smoking cessation rates^13^. This lack of correspondence might imply that PHW impact smoking cessation through mechanisms unrelated to searching for information in general. For example, where PHW are put on cigarette packages together with quit-line phone numbers, PHW may prompt regular smokers to call quit-lines instead of searching for information elsewhere. Studies from some countries, such as Australia and New Zealand, have shown that PHW are associated with a large increase in the number of quit-line calls^40,41^.

There is some positive evidence for an impact in France and, to a lesser extent, in the UK. The lack of consistency in the evidence for the six countries may reflect true variations in the extent to which PHW, as implemented in different countries, have affected smoking cessation rates. It has been suggested that the impact of PHW may be enhanced by the use of publicity campaigns around the implementation deadline, as in England^38^. The types of images of the PHW and their perceived averseness might influence their effectiveness^34^. The PHW used in the six countries did not differ very much in terms of coverage (Table 1), but differed in message and presentation, and thus may have differed in warning reach, salience and persuasiveness.

We should stress that PHW were not implemented with the explicit aim to stimulate people to stop smoking. The aim was to inform smokers about the health consequences of exposure to tobacco smoke, and the addictive nature of the product^42^. There is evidence from population-wide studies for the PHW to be effective in these terms. For example, a study based on the ITC data found that in Australia, in which some PHW showed that smoking can cause blindness, much more smokers know smoking can cause blindness than in countries where these pictures were not included in the PHW set^43^. Future studies on this topic could employ the methods used in this paper to investigate changes in online searching for the specific diseases or other topics covered by the warnings.

## CONCLUSIONS

In line with other reviews, a recent review^21^ based on 37 studies about PHW, concluded that there is widespread evidence that PHW are more effective in inducing people to stop smoking compared to text-only warnings. Based on this, we expected that the implementation of PHW would be associated with increased internet searches for smoking cessation. However, we found no consistent support for such a positive impact. While this finding is not necessarily in conflict with those of other studies, it does raise issues for further research. First, more studies employing an interrupted time-series design are needed to assess the precise magnitude and duration of population-wide impacts of PHW. Second, comparative studies are needed to study how this impact may depend on the way in which PHW are designed and implemented in specific countries. Third, in-depth studies on both smoking cessation and its precursors are needed to assess the ways PHW (and associated measures) may affect smoking cessation rates in national populations. Finally, further methodological development is needed, including the use of data on internet searching, not only to assess the impact of PHW, but also to pave the way for future studies on population-wide impacts of plain packaging.
