# Supplementary material for: Did the introduction of pictorial health warnings increase information seeking for smoking cessation?: Time-series analysis of Google Trends data in six countries
Source: Tob Prev Cessat. 2019 Aug 2;5:27. doi: 10.18332/tpc/111130 (PMC7205058; doi:10.18332/tpc/111130)
Supplement: Supplementary file 1 [file TPC-5-27-s1.pdf]

Supplementary Online Material

Table A1. Sensitivity analysis: non-transformed effects of the implementation of PHW on the RSV in three periods with a standard ARIMA model (0,1,1)(0,0,0) per country

	**1 month after implementation**		**2 – 3 months after implementation**		**4 – 6 months after implementation**	
**Country**	**B**[1]	p	**B**	p	**B**	p
**Denmark**	0.70	0.307	-0.21	0.598	0.14	0.537
**France**	**5.56**	**0.008**	-1.32	0.287	-0.32	0.741
**Ireland**	-4.84	0.556	1.91	0.713	1.22	0.648
**Norway**	-0.61	0.930	2.31	0.605	1.98	0.303
**Switzerland**	-19.70	0.118	8.91	0.253	-6.30	0.137
**UK Stop**[2]	-0.97	0.679	1.37	0.340	-0.07	0.955
**UK Quit**[2]	1.55	0.286	-0.52	0.571	-0.04	0.953

1 Estimated increase in RSV due to the introduction of PHW. 2 For the UK, two different search terms were modelled separately: ‘Stop smoking’ and ‘Quit smoking’. Significant effects are highlighted in bold ($p < 0.05$).

Table A2. Sensitivity analysis: non-transformed effects of the implementation of PHW on the RSV in three periods with a 'deactivated' ARIMA model (0,0,0)(0,0,0) per country

	**1 month after implementation**		**2 – 3 months after implementation**		**4 – 6 months after implementation**	
**Country**	**B**[1]	p	**B**	p	**B**	p
**Denmark**	1.63	0.503	1.25	0.472	**2.88**	**0.030**
**France**	**15.40**	**0.001**	**15.70**	**< 0.001**	**10.12**	**< 0.001**
**Ireland**	-11.98	0.306	-7.92	0.341	-8.97	0.189
**Norway**	-4.57	0.531	-2.31	0.656	2.88	0.500
**Switzerland**	-15.67	0.368	-3.31	0.789	-7.28	0.475
**UK Stop**[2]	-4.50	0.227	-0.14	0.956	-2.09	0.316
**UK Quit**[2]	-4.06	0.126	-4.31	0.015	**-5.33**	**< 0.001**

1 Estimated increase in RSV due to the introduction of PHW. 2 For the UK, two different search terms were modelled separately: 'Stop smoking' and 'Quit smoking'. Significant effects are highlighted in bold ($p < 0.05$).

Figure S1. Google trends a) seasonally adjusted RSV and b) ARIMA model (0,1,1)(1,0,0) for Denmark

Denmark - Seasonally adjusted RSV

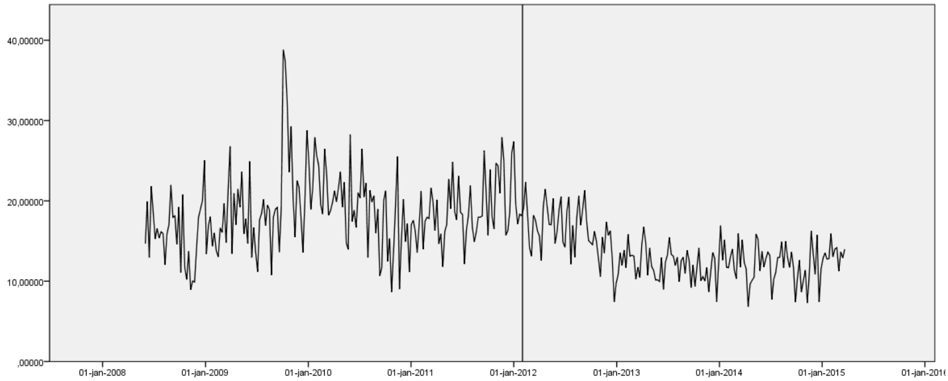

Denmark - ARIMA model

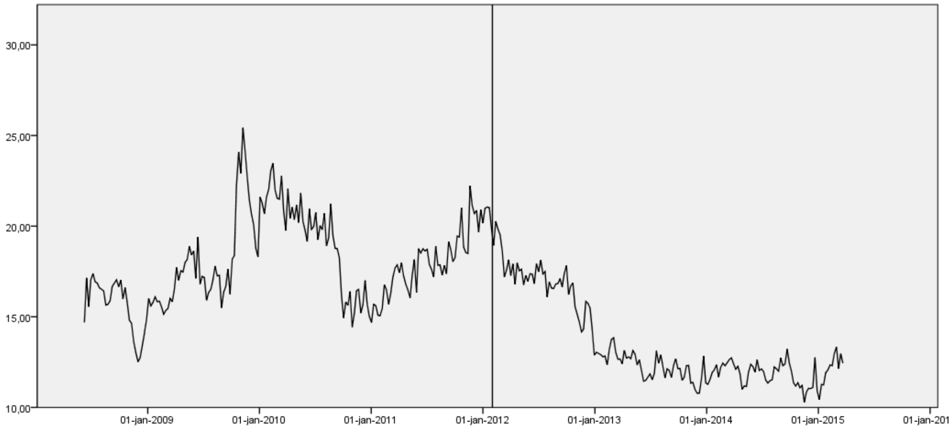

Figure S2 Google trends a) seasonally adjusted RSV and b) ARIMA model (0,1,1)(0,0,0) for France

France - Seasonally adjusted RSV

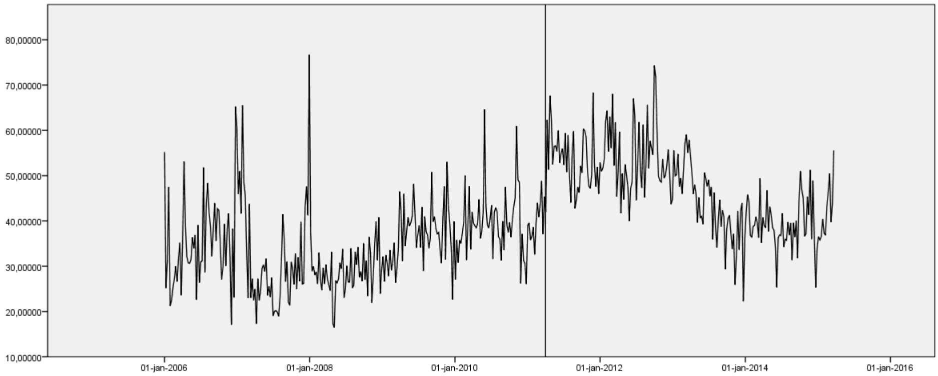

France - ARIMA model (0,1,1)(0,0,0)

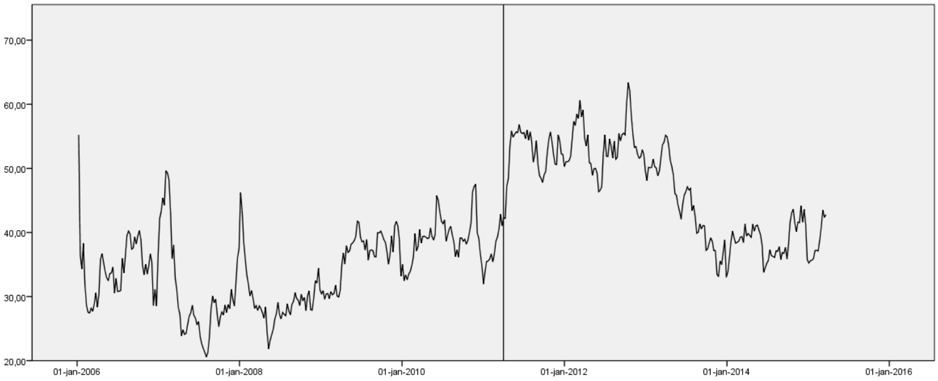

Figure S3. Google trends a) seasonally adjusted RSV and b) ARIMA model (0,1,1)(1,0,0) for Ireland

Ireland - Seasonally adjusted RSV

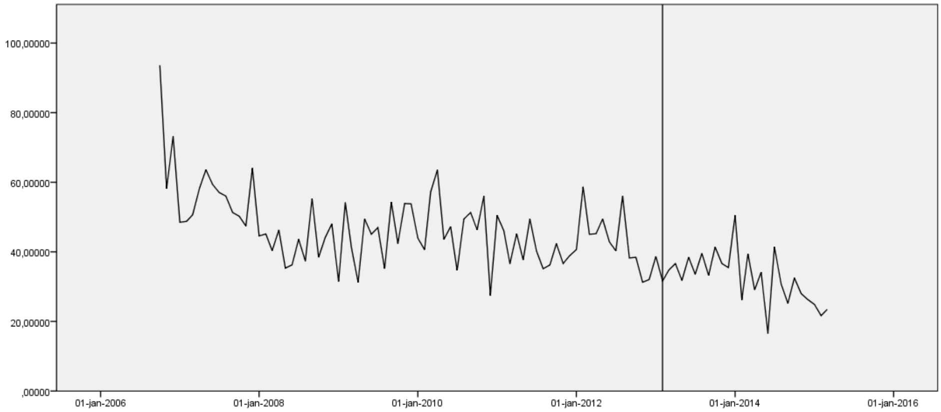

Ireland - ARIMA model (0,1,1)(0,0,0)

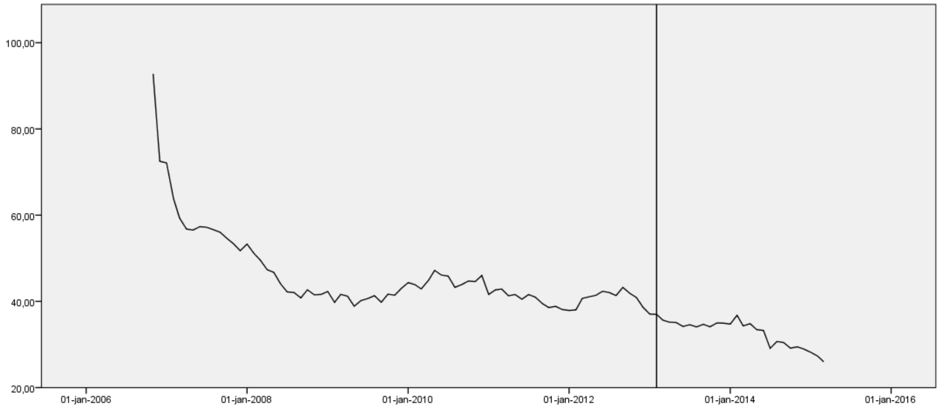

Figure S4. Google trends a) seasonally adjusted RSV and b) ARIMA model (1,0,0)(0,0,0) for Norway

Norway - Seasonally adjusted RSV

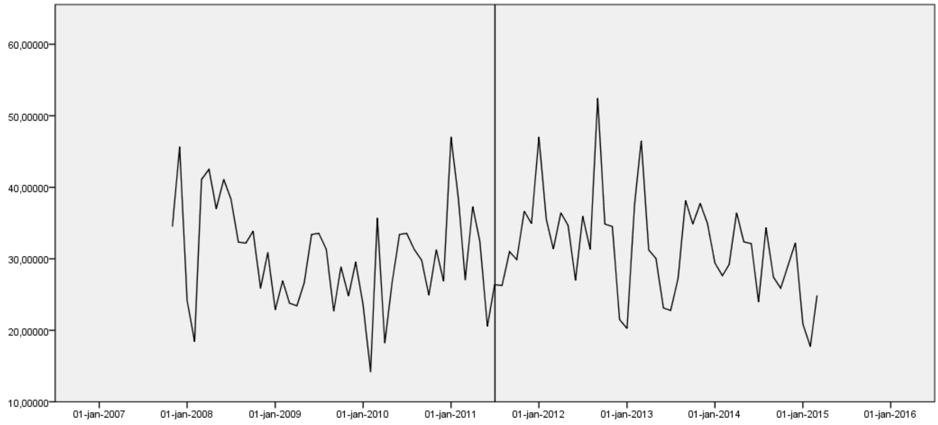

Norway - ARIMA model (1,0,0)(0,0,0)

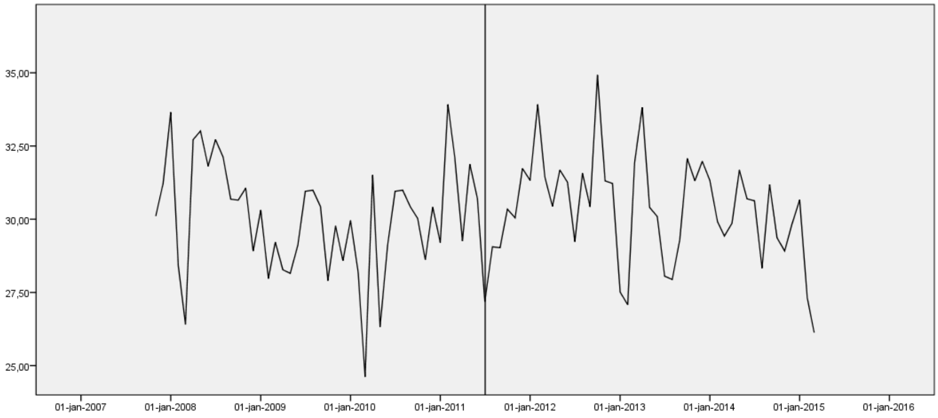

Figure S5. Google trends a) seasonally adjusted RSV and b) ARIMA model (0,1,1)(0,0,0) for Switzerland

Switzerland - Seasonally adjusted RSV

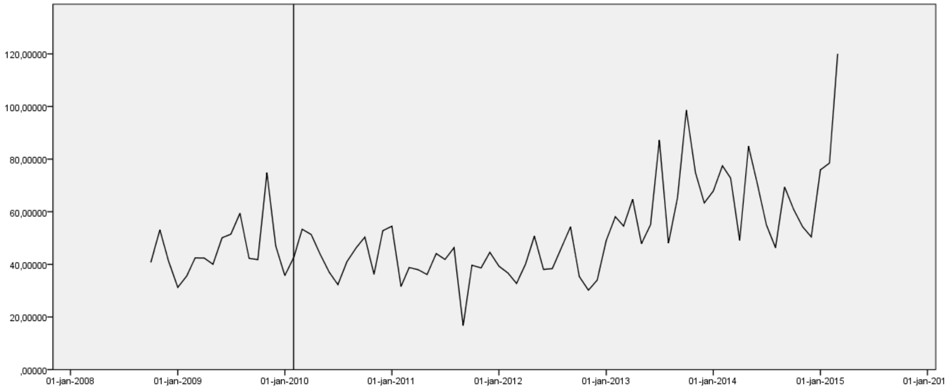

Switzerland - ARIMA model (0,1,1)(0,0,0)

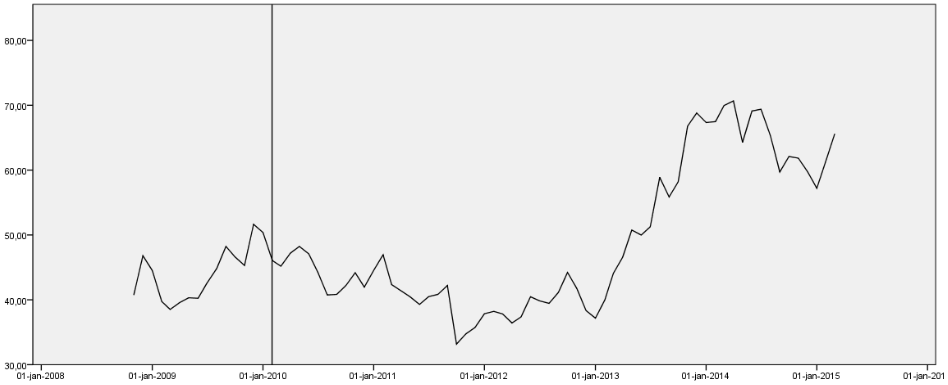

Figure S6. Google trends a) seasonally adjusted RSV and b) ARIMA model (1,1,9)(0,0,0) for UK “stop smoking”

UK (Stop smoking) - Seasonally adjusted RSV

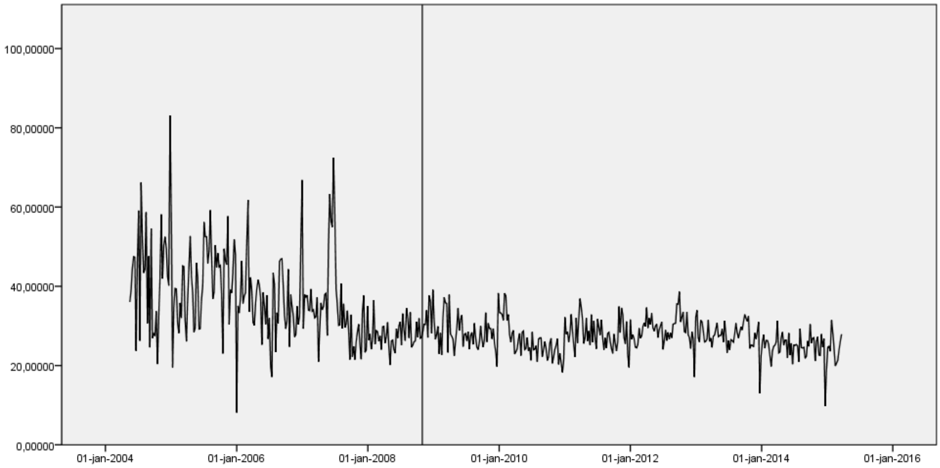

UK (Stop smoking) - ARIMA model (1,1,9)(0,0,0)

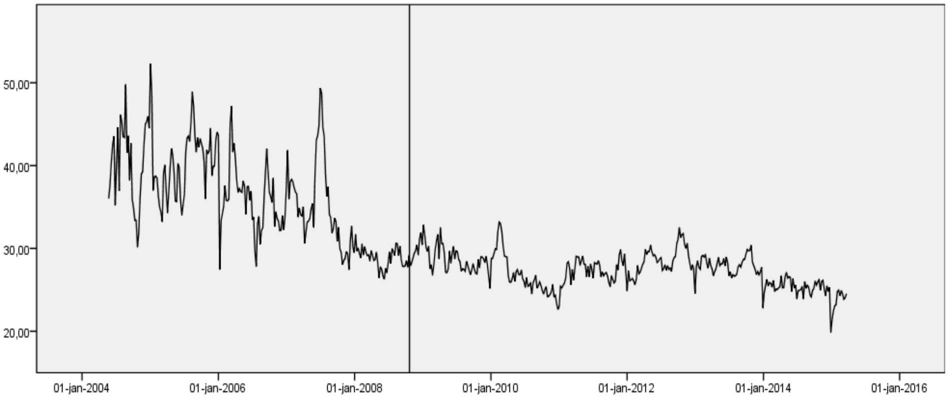

Figure S7. Google trends a) seasonally adjusted RSV and b) ARIMA model (0,1,1)(1,0,1) for UK "quit smoking"

UK (Quit smoking) - Seasonally adjusted RSV

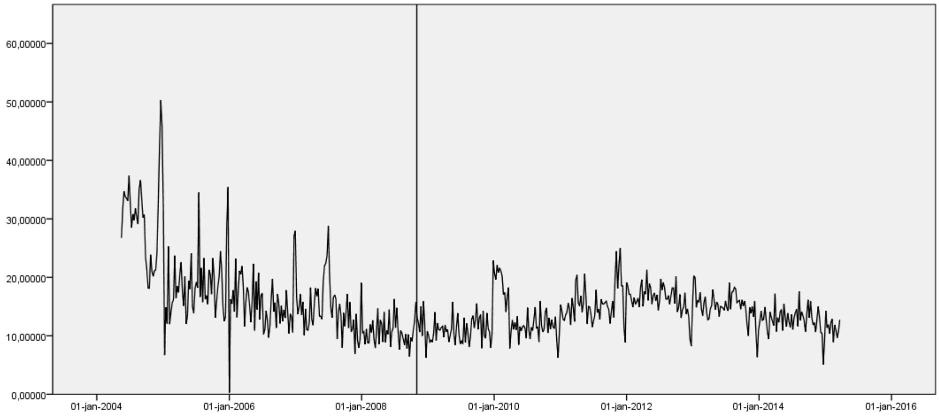

UK (Quit smoking) - ARIMA model (0,1,1)(1,0,1)

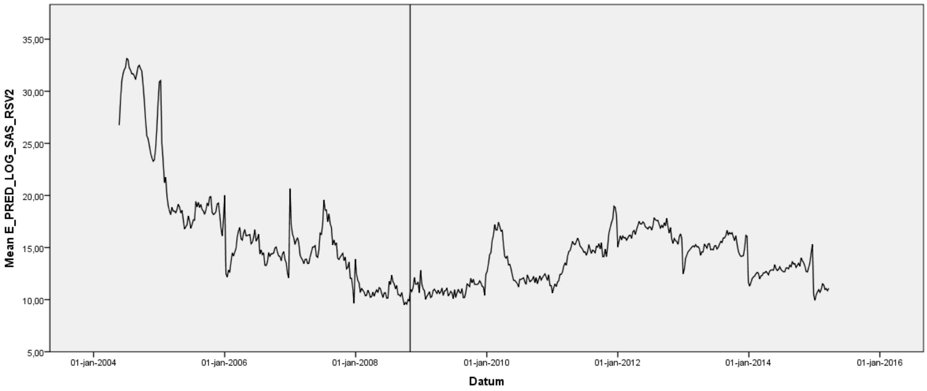

Appendix Figure S8 RSV for search terms indicative of the introduction date of PHW

Denmark - RSV of the search term 'Cigaretpakker'

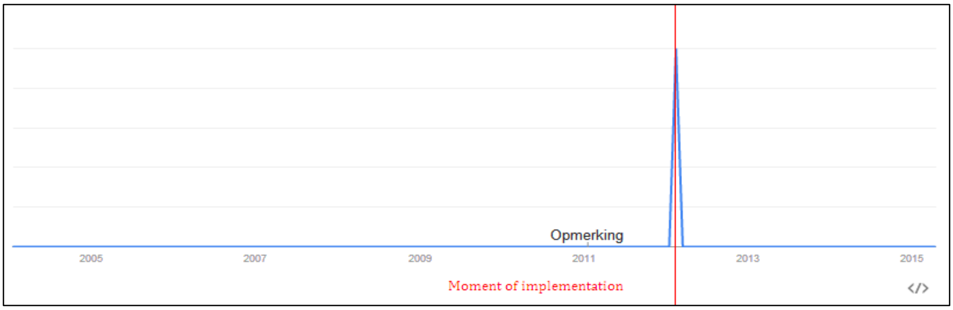

France - RSV of the search term 'Images cigarette'

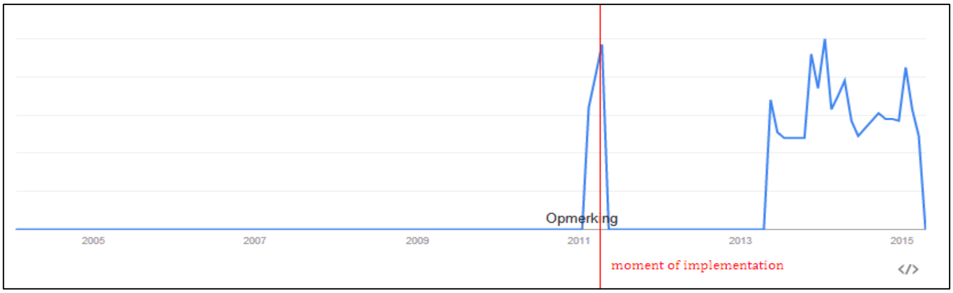

UK - RSV of the search term 'Pictures cigarette'

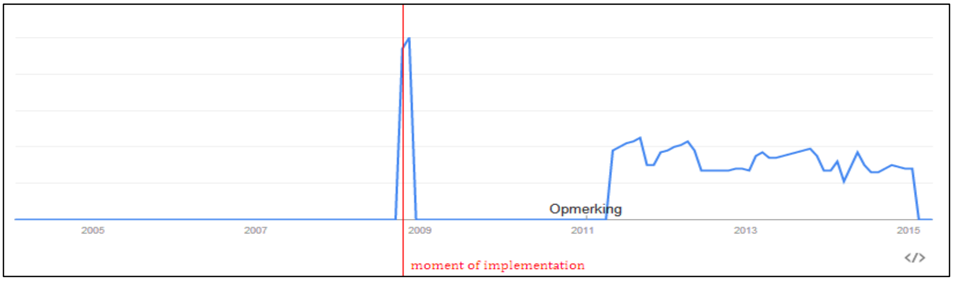

Note: No relevant data could be extracted from Google Trends data available for Ireland, Norway and Switzerland.

Figure S9 Graphics of the RSV's of random 'how to' search terms worldwide

RSV of the search term 'How to clean'

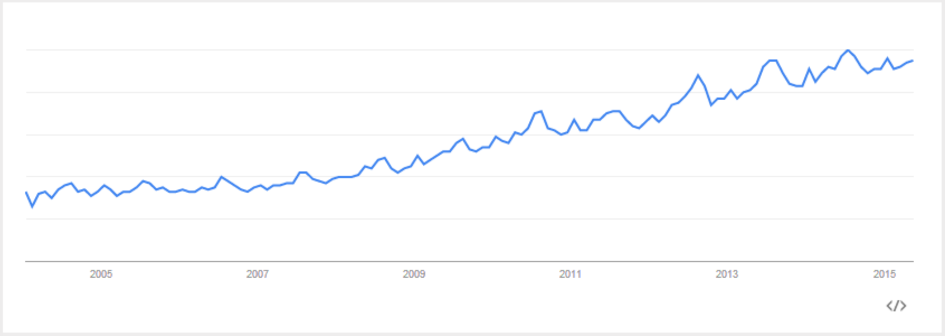

RSV of the search term 'How to Google'

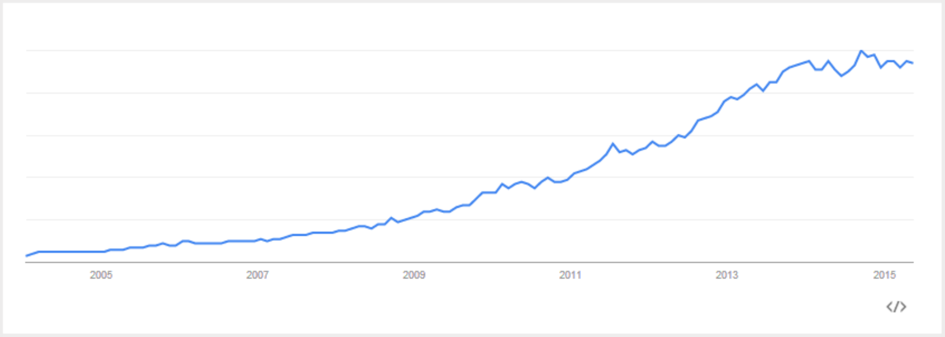

RSV of the search term 'How to draw'

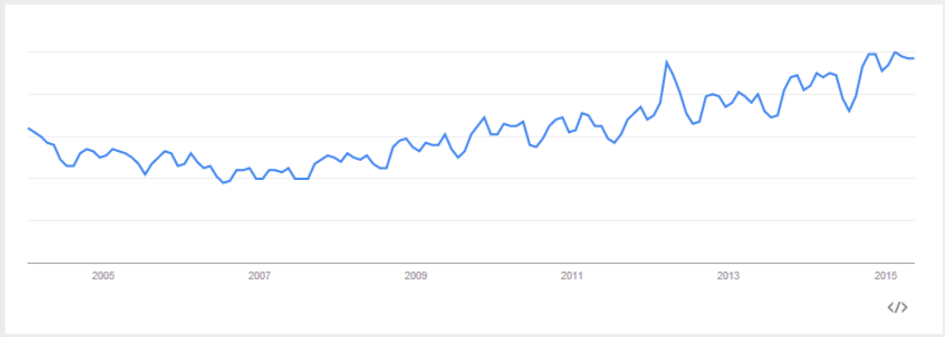

© 2019 Kunst E.A.
